# Bristow-Latarjet Technique: Still a Very Successful Surgery for Anterior Glenohumeral Instability - A Forty Year One Clinic Experience

**DOI:** 10.3889/oamjms.2015.066

**Published:** 2015-06-09

**Authors:** Vilson Ruci, Artid Duni, Alfred Cake, Dorina Ruci, Julian Ruci

**Affiliations:** 1 *Department of Orthopaedics, Faculty of Medicine, University of Medicine, Tirana, Albania*; 2 *Orthopaedics, Trauma Hospital Centre, Tirana, Albania*; 3 *Rheumatology and Rehabilitation, Mother Teresa University Hospital, Tirana, Albania*

**Keywords:** Bankart lesion, shoulder, Bristow, Latarjet, instability

## Abstract

**AIM:**

To evaluate the functional outcomes of the Bristow-Latarjet procedure in patients with recurrent anterior glenohumeral instability.

**PATIENTS AND METHODS:**

Personal clinical records of 42 patients with 45 operated shoulders were reviewed retrospectively. Patient age at time of first dislocation, injury mechanism, and number of recurring dislocations before surgery were recorded. The overall function and stability of the shoulder was evaluated.

**RESULTS:**

Thirty five (78%) of the scapulohumeral humeral instabilities were caused by trauma. The mean number of recurring dislocations was 9 (95% confidence interval [CI], 0–18); one patient had had 17 recurrences. Mean follow-up 46 months (95% CI, 16-88). No dislocation happened postoperatively. Four patients have fibrous union (9%). Only two had clinical sign of pain and discomfort. One of them was reoperated for screw removal with very good post-operative result. The overall functional outcome was good, with a mean Rowe score of 88 points (95% CI, 78–100). Scores of 27 (64%) of the patients were excellent, 9 (22%) were good, 4 (9.5%) were fair, and 2 (4.5%) were poor.

**CONCLUSION:**

The Bristow-Latarjet procedure is a very good surgical treatment for recurrent anterior-inferior instability of the glenohumeral joint. It must not be used for multidirectional instability or psychogenic habitual dislocations.

## Introduction

The glenohumeral joint is the most mobile in humans; its wide range of movement and the relatively loose anterior-inferior recess increases the risk for anterior dislocation or subluxation. 50% of all joint dislocations involve the shoulder, mostly young males. When the first dislocation occurs under 20 years old the risk for recurrent instability increases to 90%. In persons older than 40 years of age, the incidence drops sharply to 15-20 %. The majority of all recurrences occur within the first 2 years after the first traumatic dislocation [1]. Surgical stabilization of the glenohumeral joint is indicated when recurrent instability causes discomfort [2].

Coracoid osteotomy and transfer to the glenoid along with short head of biceps and coracobrachialis tendon, secured with screws were first described by Latarjet in 1954 and subsequently Helfet in 1958 [4, 5].

In reality, Helfet, did not use the screw and only sutured the bone fragment to the anterior muscular wall. Multiple modifications have been made to this original description [5, 12, 13], but the essential result is a transferred coracoid bone block reinforcing the anterior inferior glenoid margin.

Today, the Bristow- Latarjet procedure with detachment of the tip of the coracoid and its transfer to the glenoid is routinely used as a safe manner to treat anterior glenohumeral instability.

**Figure 1:**
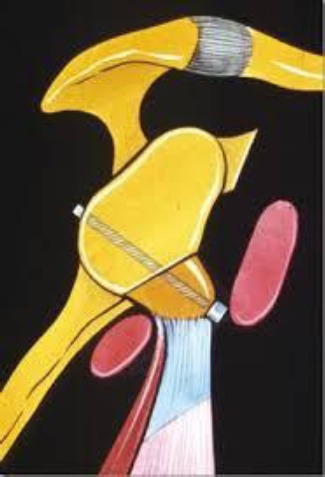
*Coracoid transfer anterior to glenoid rim fixed with a single screw*.

We intend to evaluate the functional and clinical outcome of the Bristow-Latarjet procedure in patients with glenohumeral instability treated surgically.

## Patients and Methods

The Bristow-Latarjet procedure has been used in our clinic since 1975 for patients with recurrent anterior glenohumeral instability. There were 85 patients with 92 surgeries (eight patients underwent bilateral surgeries at 2-3 years interval), performed. Available records of patients who underwent this repair between January 1997 and December 2013 inclusive were retrospectively reviewed. Only 42 patients with 45 surgeries fulfilled the criteria for our designed study. Only 2 patients (5%) were females the rest of 42 patients including four bilateral cases were adult males. Patient data including age of patient on first dislocation, injury mechanism, and events of recurring dislocations before surgical repair, time of surgery, follow-up period, and complications were recorded. Mean follow-up was 46 months (16-88).

**Figure 2:**
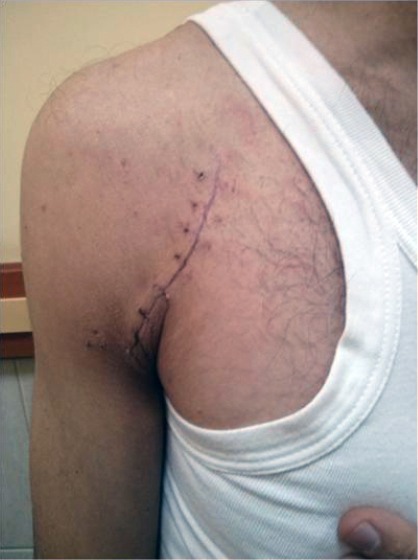
*Two weeks after surgery 35 years old patient*.

The operation started with a standard axillary incision about 8 cm (Fig. 2), thereafter the coracoideus was exposed and the musculocutaneus nerve is protected. It is of major importance to keep the insertion of the tendon of the pectoralis at the base of the coracoid process. The remaining short head of biceps tendon and coracobrachialis muscle must accompany the bone fragment. A central drill hole was placed in the coracoid tip using a 3.2-mm drill to facilitate fixation. An osteotomy of the coracoid was performed approximately 1.5-2 cm distal to the tip depending from the bone structure and pattern of coracoids process. The osteotomy can be made with an oscillating saw or with a good slightly curved 15 mm osteotome. We have used both with excellent cut and with no complication from fractured coracoids process.

In the last 5 years the senior author (V.R) started to use a modified drilling of coracoid process, making first the osteotomy and after that drilling the fragment in a retrograde fashion from the spongious surface directed to the tip of coracoids, in order to have a perfect hole in the inner surface of the graft which will be in contact with the denudated scapular inner surface. Making this modification we have seen no one case after 15 consecutive operations with fibrous union or pseudoarthrosis between fragments.

The original description of Latarjet insists in putting the coracoids in *“lying”* position and fixing with one or preferably two screws, in order to avoid non-union of coracoid fragment with the inner surface of scapula.

**Figure 3:**
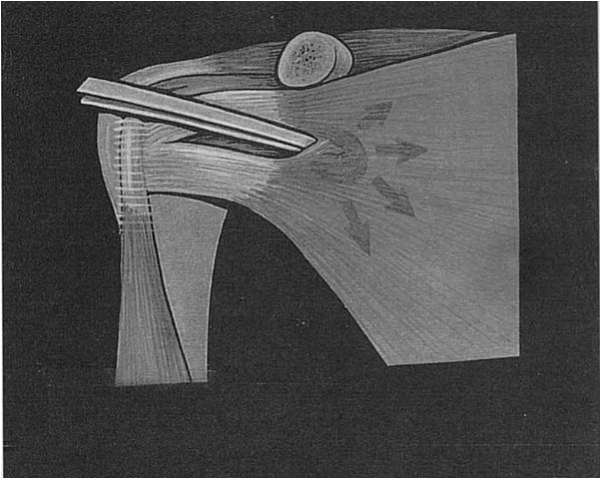
*Horizontal split of subscapularis muscle*.

We prefer to put the graft in *“standing”* position and fixing with one screw, providing a good denudated scapular surface and cancellous coracoid. We have seen no fibrous union and no bone fracture. The only difficult momentum may be the possible rotation of the coracoid piece, which can be eliminated by a stand grasping in the last phase. With the arm in external rotation, the tendon of the subscapularis was horizontally split (Fig. 3).

**Figure 4:**
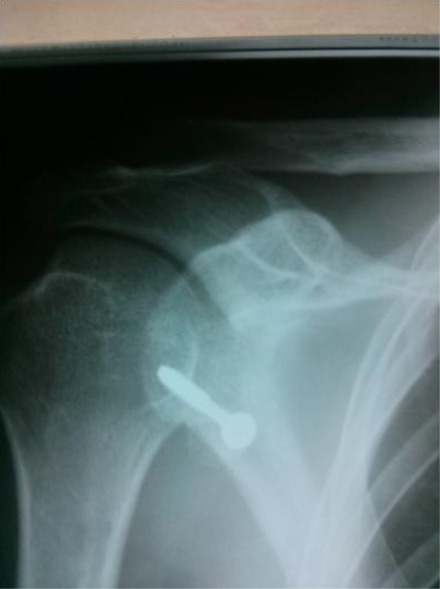
*Ideal placement for titanium screw just below the equator line*.

In our experience it was of paramount importance to respect the horizontal split avoiding the vertical incision of subscapularis tendon, although it is technically more difficult but with a very good results and early rehabilitation.

**Figure 5:**
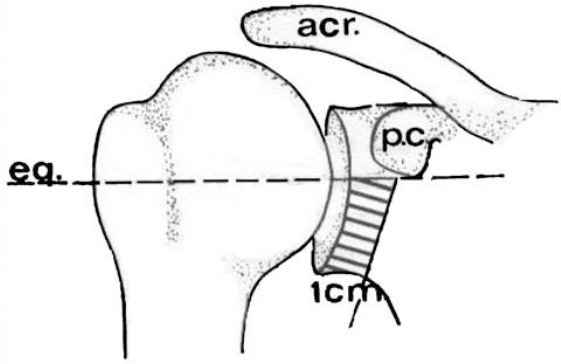
*Safe zone for screw insertion (schematic draw)*.

We recommend incising the capsule to verify accompanying intra-articular pathologies as osteochondral micro- fragments not seen in plain x-rays or the Bankart lesion. The glenoid edge must be decorticated up 2 cm medial to the glenoid, beneath the glenoid equator. A bicortical drill hole was placed in an anterior-posterior direction. The direction of drill must be perpendicular to the scapular wing and not to the operating table, thus slightly from medial to lateral posteriorly. The ideal entry point is 5 mm medial to glenoid rim and just 2 mm below the equator line (Fig. 5).

The coracoid tip was transferred and attached by a screw. Recently we use only titanium alloy screw do avoid subsequent removal for MRI purposes. Some authors used systematically washer to reinforce the fixation and to prevent bone fragmentation of coracoids. We have seen that using malleolar instead of normal screw the washer is not so important especially when the coracoids fragment in well cut and is wide enough to receive the screw. With the humerus in complete internal rotation, the tendon of the subscapularis was sutured with two or three sutures and the wound closed in layers.

The arm was immobilized in adduction and internal rotation for 15 days, and then subjected to a 3-6 weeks rehabilitation phase. Postoperatively, the range of movement at the glenohumeral joint was measured in all the three planes. The stability of the shoulder was tested using the apprehension test and Sulcus sign. The overall function and stability of the shoulder was evaluated using the Rowe score [16]. Parameters including stability, range of movement, daily function, and pain were evaluated. The final score indicated a poor surgical outcome if it was ≤50 points, fair if 51 to 74 points, well if 75 to 89 points, and excellent if 90 to 100 points. Three orthopedic surgeons evaluated postoperative radiographs of the shoulder in anteroposterior, lateral and true anteroposterior views; fusion of the transplant, position of the screw, and arthritic signs of the glenohumeral joint (classified according to Samilson and Prieto) were assessed [16].

Data were collected using an Excel worksheet and analyzed using the Statistical Package for Social. Means were calculated with 95% confidence intervals (CIs).

## Results

The mean follow-up period was 46 (95% CI, 16–88) months. Forty patients (95%) were male and 2 (5%) were female. The mean age of the patients at the time of the first shoulder dislocation was 22 (95% CI, 18–42) years. 29 (65%) involved the dominant shoulder. In 34 (75%) the first dislocation was caused by direct trauma; the remaining 11 (25%) were non-traumatic, after a forced external rotation-abduction move of the upper extremity. The mean number of recurrent dislocations was 8 (95% CI, 0–18) before surgical stabilization; one patient had had 24 recurrences. No patient had a recurrent dislocation after surgery and their mean postoperative Rowe score was 90 (95% CI, 78–100) points. Scores of 27 patients (64%) were excellent, 9 (22%) were good, 4 (9.5%) were fair, and 2 (4.5%) were poor (Table 1).

**Table 1:** Different studies reporting surgical outcomes after the Bristow-Latarjet repair

STUDY	No. of patients	Follow-up (months)	Redislocation rate	Rowe score Excellent	Rowe score Good	Rowe score Fair	Rowe score Poor
Banas et al 1993	79	103	4%	74%	11%	9	6
Singer et al 1995	14	246	0%	36%	57%	7	1
Pap et al 1997	31	31	3%	45%	39%	6	10
Hovelius et al 2004	118	182	4%	71%	15%	11	4
Matthes et al 2007	29	38	0%	59%	24%	10	7
Present study	42	46	0%	64%	22%	9.5%	4.5%

One (3%) of the latter patients achieved a Rowe score of 10 points only who had a positive Leffert test in 60º, 90º and 120º, and a positive subluxation sign despite a negative apprehension test finding. Postoperative range of movement of the repaired glenohumeral joints was good. There was a slight decrease in mean external rotation in the repaired joints compared to the opposite joint (32º [95% CI, 19º–45º] vs. 43º [95% CI, 32º–53º]. However, the difference was not clinically relevant and did not impair activities of daily living. One (2%) patient developed an aseptic necrosis of the transplant with a permanent fistula within 6 months of surgery. In the second surgical intervention, the sequestrum was removed and the transferred tendons were left in the scar tissue. The glenohumeral joint remained stable (with a Rowe score of 72) and a further surgical fusion was not carried out. At the follow-up examination, 1 patient had the screw removed already. Screw loosening was detected in 1 patient. Removal of the screw after bony fusion is recommended in such cases. 4 patients have fibrous union (9%). In all other cases complete bone union was achieved.

## Discussion

Open surgeries have been traditionally used for treatment of anterior glenohumeral instability. In 1923 Bankart first published his operative procedure placating the inferior loose capsule. In 1918 Eden was the first to suggest a bone blocking operation anterior to glenoid in order to stop mechanically the anterior migration of humeral head. In 1954 Trillat described the transfer of coracoids process anteriorly to glenoid using a nail as fixation tool.

The Bristow-Latarjet procedure was introduced in English literature by Helfet in 1958 Compared to other surgical procedures for anterior shoulder instability, it consists in an important remodeling and reorientation of the peri- articular anatomy and its possible complications remains a major challenge for even most experienced surgeons [17]. The coracoid transfer does not deal with the primary pathology of either traumatic or non-traumatic glenohumeral instability. In 1961 Mc Murray like Latarjet used screw for coracoid fixation and explained that the mechanism of joint stability was secured by the brace role of coracobrachialis and biceps tendon- muscle unit more than from “boneblock” effect of coracoid fragment. Thus the basic role of bone fragment is that of a good and safe anchor for the above two muscles which acts like dynamic stabilizers.

In recent years these techniques have regained popularity because of too many recurrences from closed Bankart or other similar soft tissue repair procedures. Also many patients, especially heavy manual workers feel a kind of loss of force after Bankart procedure which is the technique of choice for competitive athletes. Nonetheless, the major aim of surgical repair of shoulder instability is prevention of recurrent dislocations. Our patients had no recurrent dislocations after the Bristow-Latarjet repair, consistent with other studies. Outcomes of the Bristow-Latarjet procedure are comparable to other open surgical techniques [18]. Our group of patients have good postoperative range of shoulder movement, except for a slight decrease in external rotation, which did not impair activities of daily living. Moreover Latarjet procedure is a dynamic and not a statically form of preventing head dislocation. All patients could perform their jobs and sport activities almost quite normally. Despite several instances of screw loosening (two-cases), patient satisfaction was high and no neurovascular complication was observed. Severe complications after screw loosening are sporadic [18, 19]. Transplant fixation by degradable materials is still under development [23]. Arthroscopic techniques are increasingly popular for treatment of shoulder instability [22, 23]. Although the majority of glenohumeral stabilization are performed through arthroscopic assisted procedures with increasing good to excellent results this techniques needs expensive facilities and the procedure is not so easy for untrained surgeons. A learning curve is imperative even for experienced shoulder surgeons. However arthroscopic surgeries are becoming more and more accepted as the treatment of first choice in among shoulder surgeons. In 2005, a meta-analysis comparing the outcome of open and arthroscopic techniques found a more favorable outcome following open procedures in terms of recurrence and return to activity [23]. Compared to open procedures, the Mantel-Heanzel pooled odds ratio for recurrent instability after arthroscopic repair was 2.04 (95% CI, 1.27–3.29), and it was 2.85 (95% CI, 1.08–3.65) for return to activity, both of which favours open procedures.

In conclusion, the Bristow-Latarjet procedure may be a quite safe open surgical treatment for recurrent anterior glenohumeral instability, with the imperative prerequisites that the surgeon is familiar with the technique and surgical anatomy of the region. The learning curve is also very important. Best results are seen in cases operated by very experienced surgeons and after at least 15 operation performed. Although it is a non-anatomical repair, it provides very good to excellent functional results. Anterior stabilization of the glenohumeral joint by means of a modified Bristow procedure provided excellent stability in 85% of patients at long-term follow-up. Radiological findings (e.g. screw loosening, fibrous union) do not always correlate with the functional outcome and patient satisfaction. Poor results were seen in patients who reported high levels of pain and limited range of motion in the shoulder. The Bristow-Latarjet procedure remains a good method of treatment for primary anterior shoulder instability. This procedure continues to be a safe treatment option in a wide range of patients with posttraumatic anterior shoulder instability in terms of stability with good performance with regard to overall symptoms.
